# Impact of Final Phase Social Isolation and the COVID-19 Pandemic on Eating Behavior, Sleep Quality, and Anxiety Level

**DOI:** 10.3390/nu15092148

**Published:** 2023-04-29

**Authors:** Simone Gonzaga do Carmo, Júlia Paulino Teixeira Oliveira, Bruna de Almeida Aragão, Patrícia Borges Botelho

**Affiliations:** Department of Nutrition, Faculty of Health Science, University of Brasília, Campus Universitário Darcy Ribeiro, Brasília 70910-900, Brazil; simonegonzaga86@gmail.com (S.G.d.C.);

**Keywords:** COVID-19, dietary habits, eating behavior, sleep quality, anxiety level

## Abstract

The coronavirus disease 2019 (COVID-19) pandemic has resulted in economic, social, and behavioral changes in people, which may favor several long-term consequences. This study evaluated the effects of the COVID-19 pandemic on eating behavior and mental health in the final phase of social isolation. This cross-sectional study included 756 adults that completed an online questionnaire. Individuals were stratified into those who had been infected with COVID-19 (GCOV) and those who did not (GNCOV). The GCOV group had higher weight (*p* = 0.013), body mass index (BMI) (*p* = 0.005), anxiety levels (*p* = 0.040), sleep disorders (*p* = 0.009), and poorer sleep quality (*p* = 0.0028). In the GCOV, the consumption of ultra-processed foods was associated with higher anxiety levels and poorer sleep quality. A higher proportion of individuals who consumed more than five servings of in natura food was observed in the group with taste and olfactory dysfunction than in the group without. Obesity contributes to uncontrolled and emotional eating disorders, increased anxiety, and worsened sleep. Therefore, COVID-19 impaired mental health and eating behavior even in the long term. These changes were potentiated by the presence of obesity and consumption of ultra-processed foods, evidencing the importance of monitoring these individuals even after the resolution of COVID-19.

## 1. Introduction

Coronavirus disease 2019 (COVID-19) is a severe acute respiratory syndrome caused by the SARS-CoV-2 virus that has had health effects on individuals worldwide [1,2]. The COVID-19 pandemic was declared in March 2020, when restrictive measures were set forth in Brazil given the high transmissibility of the virus resulting in a range of measures to restrict movement and activities to control its spread [3].

Two years after the beginning of social distancing measures in Brazil, measures to ease these restrictions had been adopted. In May 2021, the Brazilian Ministry of Health issued guidelines for the safe return to in-person work, and in September 2021, the use of masks in outdoor areas was no longer mandatory in the country, as long as maintaining a safe distance between people. Despite the passage of time, at that moment, the weekly number of confirmed infection cases had remained high at around 118,790, and the number of deaths had reached 3176. The end of the emergence of public health in Brazil was decreed by the government in April 2022. According to the World Health Organization, until 24 March 2023, 771,071,826 individuals were infected, and 6,879,677 deaths occurred worldwide, while in Brazil, 37,145,514 cases and 699,634 deaths were confirmed [4].

The changes resulting from this social isolation at the beginning of the pandemic, such as changes in routine, closure of services, reduction in the movement of people, and decreased food availability, have resulted in various emotional, mental, and behavioral disorders [5,6,7,8]. A Metanalysis study which included studies conducted in China, Turkey, Iran, and Italy, suggests that 47% of COVID-19 patients experienced anxiety and 34% of patients experienced sleep disturbances [9]. In addition, in Brazil, 47.2% of participants in a survey reported anxiety symptoms in the period May to August 2020 [10], and 55% reported poor sleep quality between May and July 2020 [11], evidencing that both the infection and the restrictive measures influenced the emotional issues. Moreover, Taquet et al. [12] showed that individuals who had COVID-19 presented an increased risk of developing psychiatric disorders, such as anxiety and insomnia, compared to those who did not have the disease, demonstrating that the diagnosis of the disease is a factor that also can contribute to future emotional complications. However, there is still a lack of evidence if COVID-19 continues to influence the anxiety level even after recovery, as experienced emotional trauma can reflect on future behaviors.

Anxiety is one of the main factors that can lead to changes in eating behavior [8]. Individuals with anxiety tend to have worse sleep quality and to eat in response to negative emotions [13], such as stress or sadness [8]. This is often associated with higher consumption of calorie-dense, nutrient-poor foods [7,13,14] and may affect the nutritional status of individuals. Poor quality sleep has also been associated with changes in appetite hormones, increased cravings for high-fat and high-sugar foods, and a reduced ability to regulate food intake [8,15]. In Brazil, in the first five months of the pandemic, the frequency of consumption of instant meals and fast food has increased, while the consumption of fruits and vegetables has decreased [16]. In addition, these changes in food intake were also influenced by COVID-19-related disorders such as dysgeusia and anosmia, which are characterized by alteration of taste and smell [9], and an association among emotional eating, increased food intake, and stress levels were also observed [17]. It is noteworthy that these studies were conducted at the beginning of the pandemic. This way, it is not yet known if, after the flexibility of the restriction measures, the relationship between these parameters remained or was modified. 

Food choices and eating behaviors are influenced by a multitude of factors. The COVID-19 pandemic has brought about new challenges that have further impacted these factors, creating a need for a comprehensive approach to address these interconnected issues [18]. In this sense, the hypothesis of the study is that emotional and alimentary alterations that occurred at the beginning of the pandemic may also extend even after the relaxation of restriction measures and are influenced by the diagnosis of the disease, which would raise the importance of the evaluation and detection of long-term effects. 

Thus, the objective of this study was to evaluate the relationship between the COVID-19 pandemic and eating behavior, sleep quality, and anxiety levels in Brazilians in the final phase of social isolation measures. Secondary, as a specific objective, is to assess how these parameters relate to each other, as well as to evaluate the influence of olfactory and taste sequelae on these parameters.

## 2. Experimental Methods

### 2.1. Study Design

This cross-sectional observational study was conducted between September 2021 and March 2022 in Brazil. During this period, restriction measures such as mask use are no longer mandatory in some places. In addition, most workers had returned to the workplace with a suspension of the home office. The end of the emergence of public health in Brazil was decreed by the government in April 2022, and the decrees that established pandemic control measures were revoked.

Although this period corresponds to the final phase of social isolation in Brazil, face-to-face clinical research has not yet been released, and therefore, a self-reported questionnaire was used. It is worth mentioning that several studies were conducted with a self-reported questionnaire during the pandemic Al-Musharaf [1], Liboredo et al. [17], and Couthlard et al. [14]. In addition, Davies et al. [19] and Moreira et al. [20] demonstrated that the use of self-reported anthropometric measures presented a good agreement with the measured, demonstrating that this parameter can be used in research in those cases in which it is not possible to perform the measurement. With this in mind, we adopted the online questionnaire as a tool for carrying out the research.

This study was conducted according to the guidelines laid down in the Declaration of Helsinki, and all procedures involving human subjects were approved by the Ethics Committee on Human Research of the University of Brasília (UnB) (approval number: 4.956.506).

Participants aged 20 years or older were invited to participate through the dissemination of the questionnaire link via email to universities in all regions of Brazil and some health units. In addition, this was also released on social networks (Facebook, Instagram, and WhatsApp). The eligibility criteria consisted of a minimum age of 20 years, living in Brazil, and having access to the Internet to complete the online questionnaire. Pregnant and lactating women were excluded. Trained researchers conferred the number of respondents and answers received weekly, verifying whether participants met the eligibility criteria.

Initially, the study also included elderly individuals. However, due to a low response rate from this group, the elderly were subsequently excluded from the analysis. Therefore, the statistical analysis was conducted using only data from individuals aged between 20 and 59 (adults).

### 2.2. Instruments and Data Collection

The first section of the survey included an informed consent form. The questions were categorized into the following nine sections to avoid participant fatigue when filling out the questionnaire: (1) sociodemographic and anthropometric data, (2) meditation practices, (3) food consumption, (4) implications of COVID-19 infection, (5) eating behavior, (6) sleep quality, (7) effects of social isolation, (8) mental health, and (9) body image assessment. All sections were considered in this study, except Section 2 and Section 9.

### 2.3. Sociodemographic and Anthropometric Characteristics

Sociodemographic data included questions about the place of residence, sex, race, age, education level, marital status, profession and work, the composition of residents in the residence, and income.

Anthropometric data were assessed using self-reported weight and height, both current and prior to the pandemic, to calculate body mass index (BMI) and weight change. BMI was calculated as weight (kg) divided by height (m^2^). The BMI classification was performed according to the WHO: underweight (<18.5 kg/m^2^), eutrophic (18.5–24.9 kg/m^2^), overweight (25.0–29.9 kg/m^2^), class I obesity (30–34.9 kg/m^2^), class II obesity (35–39.9 kg/m^2^), and class III obesity (≥40 kg/m^2^). Weight variation was calculated by subtracting post-pandemic weight from pre-pandemic weight. 

The questionnaire specified the units of measurement in the case of numerical variables and had a generic language for a better understanding of the public.

### 2.4. Assessment of Food Consumption

The consumption pattern of minimally or in natura ultra-processed foods was assessed using the Surveillance System for Risk and Protection Factors for Chronic Diseases by Telephone Survey (VIGITEL) questionnaire [21]. VIGITEL is part of the Brazilian government’s actions to monitor the frequency and distribution of risk and protective factors for chronic noncommunicable diseases in all capitals of the 26 Brazilian states and the Federal District. The questionnaire was based on the previous day’s consumption and assessed whether there was consumption of the foods listed in the 23 groups. The markers for healthy eating were raw salad, vegetables, cooked vegetables, fresh fruit, fruit salad, milk, and yogurt. The markers of unhealthy eating were fried food, hamburgers and sausages, salty or packaged snacks, sweet or candy cookies, and sugary drinks.

The frequency of food group consumption patterns was assessed by reporting the intake of five daily servings of each group, as recommended in the questionnaire [21].

### 2.5. Diagnosis of COVID-19 and Its Consequences

Participants were questioned if they had been diagnosed with COVID-19 at any point during the pandemic. In case of a positive answer, to evaluate the effects of this disease, participants replied about the symptoms presented, time of onset of symptoms in relation to the day the questionnaire was filled out, duration, changes in senses such as taste and smell, impact on memory, sequelae, and consequences of the disease perceived to have interfered in some way with eating. For this, the following questions were asked: Were you diagnosed with covid? Did you have covid symptoms? How long have you had covid symptoms? How long did the symptoms last? Did you have a taste change? Did you have a change in smell? What sequels did you present? What are the consequences of this sequel about your diet?

### 2.6. Eating Behavior

The translated and validated Three-Factor Eating Questionnaire (TFEQ- 21) was used to assess eating behavior [22]. The questionnaire consisted of 21 questions that assessed three dimensions of eating behavior: emotional eating (EE), uncontrolled eating (UE), and cognitive restriction (CR). The EE, UE, and CR domains evaluated the tendency to consume food in an exaggerated manner as a response to emotional conditions or states and feelings, loss of control over food intake in the face of feelings of hunger or external stimuli, and dietary restrictions on weight control, respectively.

Items 1 to 20 encompassed questions with a four-point format and question 21, an eight-point numerical scale. To interpret and determine the grades in each domain, the authors’ recommendations were followed. 

The highest score in each domain (closest to 100) represented greater dysfunctional eating behavior. 

### 2.7. Quality of Sleep

Sleep quality was assessed using the Pittsburgh Sleep Quality Index (PSQI) questionnaire translated into the Brazilian population [23].

The dimensions evaluated in the questionnaire consisted of seven components: C1 (subjective sleep quality), C2 (latency), C3 (duration), C4 (efficiency), C5 (sleep disorders), C6 (use of sleep medications), and C7 (daytime dysfunction), which were scored from 0 to 3 according to the authors’ instructions. The overall PSQI score ranged from 0 to 21 points; thus, the scores obtained throughout the components were added for classification assigned as “good sleep quality”, “poor quality”, and “sleep disturbance” when the participants had final scores between 0 and 4 points, 5 and 10 points, or greater than 10 points, respectively.

### 2.8. Anxiety Level

Anxiety levels were assessed using the Beck Anxiety Inventory (BAI) questionnaire. Participants classified the intensity of their anxiety symptoms as absent, mild, moderate, or severe. To interpret the results, a scale scoring from 0 to 3 points was used, as instructed by the authors [24]. The sum of the items resulted in a score ranging from 0 to 63 points, and the final classification of symptoms was minimal, mild, moderate, or severe when the score reached 0–10, 11–19, 20–30, or 31–63 points, respectively.

### 2.9. Statistical Analysis

Analyses were performed using the Statistical Package for the Social Science (SPSS) software (version 22, 2013; IMB Corp, Armonk, NY, USA). The study population was divided into two groups: individuals who had not been infected with COVID-19 (GNCOV) and those who had been infected with COVID-19 (GCOV).

The characteristics of the sample (age, sex, region, ethnicity, level of education, marital status, profession, family composition, income, COVID-19 infection, presence of comorbidities, food intake, and BMI) and dietary patterns were assessed using descriptive analysis. Data are presented as absolute (*n*) and relative (%) frequencies for categorical variables and as mean ± standard deviation (SD) for continuous variables. 

Normality was evaluated using the Shapiro–Wilk test. To identify differences between the GNCOV and GCOV groups in anthropometric data, eating behavior, anxiety level, and sleep difficulty, analyses of covariance (ANCOVA) were performed, controlling for age and sex. To evaluate the influence of IMC and olfactory and taste disorders on these parameters within the GCOV group, ANCOVA with post hoc Sidak’s test was also performed with age, sex, presence of diabetes mellitus, and or arterial hypertension, and time of onset of symptoms of Covid as covariable. To evaluate categorical variables in relation to food intake, the chi-square test was performed. 

In view of calculating the sample size to assess statistical power, we used the model proposed by Miot, 2011 [25]. One hundred and fifty per group participants were required to establish the anxiety level differences between groups described in Tsukamoto et al. [26] (F (1) = 6.354, *p* = 0.013). An error value β of 20% and 95% confidence level (CI). Including an additional 30% as an expected drop-out rate, the sample size was determined as 195 in each group. However, we chose to keep the questionnaire open until the time of declaration of the end of the emergency of public health, thus collecting as many responses as possible. Thus, we recruited a larger number of respondents than expected by the sample calculation, having had 75% statistical power, with a confidence interval (CI) of 95% and *p* < 0.05, considering anxiety as the primary outcome. All questionnaires have good internal consistency, with Cronbach’s alpha coefficient of 0.70 for PSQI, 0.91 for BAI in our study; on the whole, the literature showed 0.73 [23] and 0.86 [24]. The domains of uncontrolled eating, emotional eating, and cognitive restriction also showed good internal consistency, with Cronbach’s alpha values of 0.85, 0.91, and 0.77, respectively, close to the values (0.85) described in the literature [22].

## 3. Results

### 3.1. Characterization of the Participants

A total of 847 participants completed the questionnaire. People who did not complete the questionnaire were under 20 years old, or were pregnant were excluded. As only 33 seniors answered the questionnaire, they also were excluded, totaling 756 participants (Figure S1). The characterization of the participants is in Supplementary Table S1.

The mean age of the participants was 30.48 ± 9.88 years. The study population comprised mostly females (81.70%, *n* = 618) (Supplementary Table S1). Most individuals (63.20%, *n* = 478) reported working at the time of the study, with 20.6% (*n* = 152) being healthcare professionals, 22.00% (*n* = 162) administrative service workers, and 27.10% (*n* = 200) students. COVID-19 was reported by 40.10% (*n* = 303) of the participants.

Assessment of nutritional status by BMI showed that 59.80% (*n* = 452) of participants were classified as eutrophic and 34.50% (*n* = 261) as overweight or obese. Although emotional eating, uncontrolled eating, and restrictive eating have presented an average of 42.90 ± 22.60, 39.56 ± 28.02, and 34.60 ± 20.36, respectively, a considerable percentage of people with dysfunction of food behavior at the end of social isolation was still observed. Considering a score greater than 50 as indicative of diet dysfunction, 22% of individuals presented uncontrolled eating, 37.6% emotional eating, and 45% restrictive eating. The studied population presented an average score that reflects mild anxiety (11.37 ± 9.63). However, 42.3% of the population studied had some degree of anxiety (mild, moderate, or severe). In addition, the average score for sleep quality indicated poor sleep quality (6.70 ± 3.47).

### 3.2. Relationship of COVID-19 and Factors of Eating Behavior and Mental Health

The participants were asked about the time of onset of symptoms, and 24.8% (*n* = 67) reported the presence of infection symptoms within one month of the questionnaire completion date, while 20% (*n* = 54) and 53% (*n* = 153) reported the presence of symptoms within a period of two to seven months and more than 12 months, respectively.

After adjusting for age and sex, no differences were observed in pre-pandemic weight, ponderal variation, or eating behavior between the groups (*p* > 0.05). However, ANCOVA revealed that individuals who have had COVID-19 at any point during the pandemic currently present higher weight, higher BMI, greater anxiety levels, and poorer sleep quality compared to those who have never had COVID-19. These findings suggest that COVID-19 may have long-term impacts on an individual’s physical and mental health beyond the acute illness phase (Table 1).

When the consumption of at least five servings of in natura or minimally processed food was evaluated between the groups, the GCOV presented a lower percentage of individuals who reported adequate consumption (*p* = 0.039).

Taste and smell dysfunction was not associated with anthropometric measurements, eating behavior, anxiety, or sleep quality (*p* > 0.005) (Supplementary Table S2). However, a higher proportion of individuals who consumed more than five servings of in natura was observed in the group with taste and olfactory dysfunction than in the group without.

### 3.3. Relationship between the Consumption of Processed and in Natura Foods, Eating Behavior, and Mental Health of Individuals Diagnosed with COVID-19

The prevalence of individuals who were infected with COVID-19 was 40.10%. Of these, 20.10% and 89.80% had a high consumption of ultra-processed foods and in natura foods, respectively.

Those individuals who had a higher consumption of ultra-processed food had worse sleep quality and higher levels of anxiety (*p* = 0.013) when adjusted by age and sex. However, this significance remained only for sleep quality when the model was adjusted for symptom onset time and the presence of chronic diseases (Table 2). In addition, anxiety did not influence eating patterns in the final phase of social isolation (Table 3).

### 3.4. Relationship between BMI, Eating Behavior, and Mental Health of Individuals Infected with COVID-19

Among individuals infected with COVID-19, higher BMI values were associated with higher eating behavior scores, anxiety levels, and sleep scores (Table 4).

Overweight and obese individuals had higher scores in uncontrolled eating [F(1219) = 8.385; *p* < 0.01] and emotional eating [F(3223) = 11.883; *p* < 0.01] than eutrophic and underweight individuals. Low-weight individuals had the lowest cognitive restriction scores [F(3292) = 7.913, *p* < 0.01]. A higher level of anxiety [F(3219) = 6.673; *p* < 0.01] and worse sleep quality [F(3183) = 4.076; *p* < 0.01] were also observed among overweight and obese individuals than among eutrophic individuals.

### 3.5. Relationship between Onset and Duration of Symptoms, Eating Behavior, and Mental Health of Individuals Infected with COVID-19

We performed a hierarchical multiple regression analysis to investigate the ability of onset and duration of symptoms to predict eating behavior scores and mental health.

The duration of symptoms explained 2.9% of the total variance in the BAI score so that having more than 15 days of symptoms increases by 4.390 units the anxiety score compared to less than 15 days of symptoms. The other parameters were not influenced by the duration of symptoms (Table 5).

The onset of symptoms explained 1.5% of the total variance in BAI score so that having more than 12 months of diagnosis and onset of symptoms reduces by −0.773 units the anxiety score compared to less than one month. The other parameters were not influenced by the onset of symptoms (Table 6).

## 4. Discussion

The present study showed, in an unprecedented way, the association of COVID-19 with the eating behavior, anxiety level, and sleep quality of adult participants in the final phase of pandemia.

Our results showed the effects after 18 months of the pandemic, given the time of application of the questionnaires when flexibilization measures had already resumed. During the survey period, 63.20% of participants had returned to work. Although the routine of many individuals was returning to normality, the consequences resulting from social isolation in the first months, as demonstrated in several studies [3,13,17,27,28,29,30,31,32], were perpetuated in the long term even after the return of activities.

Among the changes noted was the effect on the body mass of individuals. In our study, an increase in body weight was noted in 48.20% of the individual's GNCOV (*n* = 158) and 48.80% (*n* = 126) of GCOV during the pandemic, and a reduction in body mass in 28.00% (*n* = 92) and 30.60% (*n* = 79) of GNCOV and GCOV individuals, respectively. Weight change was also observed in France, where 35% of the individuals evaluated reported weight gain (1.8 kg) after two months of social isolation, and 23% reported a reduction (2 kg) [33]. Moreover, higher body mass and mean BMI values were found among the GCOV subjects. This may be explained by the fact that being overweight and obese are risk factors for COVID-19. The inflammatory state is induced by excess adipose tissue and the increased expression of angiotensin-converting enzyme-2 (ACE-2) receptor, used by SARS-CoV-2 to enter the cell [2].

It is noteworthy that the variations in weight and BMI were calculated from the self-reported weight before the pandemic, that is, 18 months ago, and the current weight, which may be influenced by memory. In addition, it is important to note that although weight and BMI are not isolated analysis parameters because they do not predict the distribution and adequacy of body mass, the changes reported by individuals can be related to changes in body composition, lifestyle, eating habits adopted during the pandemic, and the emotions involved [6].

Among the emotional frameworks, COVID-19 [29], overweight, and obesity contribute to higher levels of anxiety [29,34]. Since the initial moment of the pandemic, studies have evaluated the effect of COVID-19 on anxiety levels in the general population. Many of the initial changes were related to the consequences of social isolation and uncertainty regarding the disease [13]. Thus, it was expected that with the control of the COVID-19 outbreak, mental health-related parameters would improve, which was not observed in the present study. Individuals in the GNCOV group showed minimal symptoms (10.62), and those in the GCOV group (12.14) had mild symptoms (*p* = 0.040). Moreover, only those more than 12 months of diagnosis and onset of symptoms had a lower anxiety score at −0.77 units. Individuals who had COVID-19 in Wuhan, China, also continued to experience anxiety symptoms even six months after the onset of symptoms [35], reinforcing the idea that the emotional aspects involved in the disease may compromise the anxiety level of individuals in the long term. Higher BMI values in individuals diagnosed with COVID-19 were also associated with higher anxiety scores and symptoms (*p* < 0.001) regardless of the time of onset of symptoms and the presence of chronic diseases. In addition, increased uncontrolled eating and emotional eating scores were observed in overweight and obese individuals when compared to eutrophic individuals [35].

Thus, although an anxiety reduction over time was expected, this reduction occurred only after one year, demonstrating that the reaction and way of dealing with trauma and difficult situations can vary between countries and should therefore be considered and evaluated in health services even after the end of the pandemic. Moreover, the onset of symptoms explained only a small percentage of the variation of anxiety, which demonstrates that weight can be the trigger for these dysfunctional behaviors.

In addition to anxiety and dysfunctional food behavior, overall sleep quality was rated as poor (6.70 ± 3.47). A cohort study conducted in Brazil showed worsening sleep scores when comparing individuals in the period from 2010 to 2014 (5.0 ± 3.3) and again in June 2020 (5.7 ± 3.8; *p* < 0.01), especially in individuals who remained in quarantine during the pandemic (6.1 ± 3.9) compared to those who did not (5.0 ± 3.5; *p* < 0.01) [36]. Individuals from the GCOV had worse scores for sleep quality (*p* = 0.013) and for the components of sleep efficiency and sleep disorders compared with those from the GNCOV, which may be justified by the presence of disease symptoms, as well as anxiety due to treatment and possible sequelae. This assumption was reinforced by a meta-analysis that evaluated data from individuals with COVID-19 in 13 countries and demonstrated a negative effect on sleep [37].

The mechanism by which COVID-19 contributes to sleep-related changes and psychiatric consequences may be multifactorial, related to the infection itself and the immune response, including the direct effects of the virus, medication use, disease severity, and social isolation [8,35]. Sleep quality was also impaired in overweight and obese individuals compared to eutrophic individuals (*p* = 0.008), which can be explained by a probable alteration in the respiratory physiology of these individuals, which is compromised by excess adipose tissue [38].

Food intake is another factor that may have a bidirectional relationship with emotional aspects, as carbohydrate consumption is likely related to mental health, especially anxiety disorders, due to the influence of sugars on neuroinflammation in the hippocampus [39]. In a meta-analysis, the consumption of ultra-processed foods, which are rich in carbohydrates, was associated with anxiety and was used to mitigate stress-related anxiety during a pandemic. However, improvements in eating patterns during social isolation have also been reported [34]. Most individuals assessed in our study (55.80%; *n* = 422) had adequate consumption of in natura foods, which may be due to time, intention to increase immunity, thus protecting themselves from infection, and adaptation to social isolation.

The frequency of consumption of five or more servings of ultra-processed foods (18.30%; *n* = 138) among respondents was lower than that reported by SMAIRA et al. [40] in a study conducted with women in Brazil in 2020 (20.8%). However, the frequency of individuals consuming in natura foods in the GNCOV (93.60%; *n* = 398) was higher than that in the GCOV (89.90%; *n* = 272). When we consider the assessment of consumption among the individuals in the GCOV (*n* = 303), those who presented olfactory dysfunctions presented higher consumption than individuals without dysfunction (*p* < 0.05). The fact that the individual experienced taste alterations may have contributed to greater health care after recovery. However, anxiety did not influence food choices in the final phase of restrictive measures of social isolation, which may have occurred during the initial phases of the pandemic [39,41]. One hypothesis is that fear of reinfection and the search for improved immunity may have overridden the effects of anxiety, thereby providing healthier patterns. In addition, individuals were no longer in social isolation, which may have contributed to the lower effect of anxiety on food choices.

Although present in a smaller proportion of the population, high consumption of processed or ultra-processed foods also implied higher emotional eating, uncontrolled eating, and anxiety scores. The palatability of foods rich in sugar and sodium, such as ultra-processed foods, can promote feelings of comfort and contribute to increased intake. Additionally, exacerbated and frequent increases in serious glucose levels lead to greater irritability and anxiety. In contrast, Coulthard et al. [14] found a relationship between increased consumption of high-calorie density foods in women with higher levels of emotional eating, higher BMI values, and anxiety levels. The authors associated emotional eating in the period prior to the pandemic with a higher intake of high-caloric density snacks and lower consumption of fruits and vegetables [14]. Although this study identified a reduction in emotional eating throughout the pandemic, habits persisted during this period.

### Limitations and Strength

Our study has some limitations that should be considered. Given the unprecedented nature of the pandemic, our study did not have the objective of monitoring the evaluated parameters; thus, the type of study adopted made it impossible to make a causal inference. In addition, depression and eating disorders, recognized as confusing variables, were not evaluated in this study. The incomplete filling of data and the possible memory bias in reporting data prior to the pandemic can also be highlighted. However, this bias does not invalidate the results obtained, as previous studies have shown a high correlation between measured and self-reported data. Our sample did not represent the Brazilian population, given the predominance of females, higher education, and distribution among regions, a consequence of the sampling method, which was mainly through social media, as in other studies conducted during this period.

On the other hand, our study allowed us to identify long-term consequences that the pandemic caused in mental health and eating behavior. Social isolation plays a significant role in individuals’ behaviors. It is important to note that the increase in scores related to eating behavior, anxiety, and sleep was related to the worsening of the greatest dysfunctions. In addition, most studies evaluated the effects at the beginning of the pandemic, where the scenario was different from the moment of our study. Although it still occurred at a time of social isolation, people were already vaccinated, the measures became less restrictive and had a greater tendency to return to normality.

## 5. Conclusions

In conclusion, the COVID-19 pandemic has had significant and lasting effects on people’s eating behavior and mental health, as demonstrated by this study. The findings suggest that individuals who have been infected with COVID-19 are at higher risk of experiencing adverse outcomes, such as increased weight, BMI, anxiety levels, sleep disorders, and poorer sleep quality. The consumption of ultra-processed foods was found to be associated with higher anxiety levels and poorer sleep quality in this group. Furthermore, taste and olfactory dysfunction were associated with increased consumption of in natura food.

Obesity was also found to contribute to uncontrolled and emotional eating disorders, increased anxiety, and worsened sleep, highlighting the need for continued monitoring and support for individuals who have been infected with COVID-19 and are struggling with obesity.

Given the potential long-term consequences of the pandemic on mental health and eating behavior, it is important to prioritize interventions that can promote healthy behaviors and prevent adverse outcomes. These may include promoting healthy eating habits, regular exercise, and access to mental health services. Overall, the findings of this study underscore the need for ongoing research and intervention efforts to address the mental and physical health consequences of the COVID-19 pandemic.

## Figures and Tables

**Table 1:** Impact of COVID-19 on anthropometric data, eating behavior, anxiety level, sleep quality, and eating pattern.

	GNCOV (*n* = 425)		GCOV (*n* = 303)			
	Mean (SE)	*n*	Mean (SE)	*n*	F	*p*
Pre-pandemic weight (kg)	65.81 (0.86)	299	67.78 (0.97)	232	2.29	0.131
BMI pre-pandemic (kg/m^2^)	23.93 (0.28)	299	24.73 (0.32)	232	3.59	0.058
Current weight (kg)	66.20 (0.73)	395	69.01 (0.87)	277	6.15	0.013
Current BMI (kg/m^2^)	24.02 (0.24)	395	25.06 (0.28)	277	7.92	0.005
Weight gain (%)	48.20 *	158	48.80 *	126	-	0.329
Weight loss (%)	28.00 *	92	30.60 *	79		
Weight variation (kg)	0.69 (0.42)	314	0.59 (0.48)	241	0.02	0.876
UE	34.30 (0.99)	417	34.74 (1.21)	287	0.08	0.776
EE	39.10 (1.33)	415	39.49 (1.60)	287	0.04	0.849
CR	41.70 (1.07)	411	44.80 (1.28)	285	3.46	0.063
BAI	10.62 (0.47)	395	12.14 (0.54)	277	4.25	0.040
PSQI	6.15 (0.18)	351	6.86 (0.22)	229	6.20	0.013
C1	1.06 (0.04)	351	1.16 (0.05)	229	2.52	0.113
C2	1.28 (0.05)	351	1.38 (0.06)	229	1.63	0.203
C3	0.72 (0.04)	351	0.81 (0.05)	229	2.04	0.154
C4	0.33 (0.04)	351	0.50 (0.05)	229	6.83	0.009
C5	1.09 (0.028)	351	1.19 (0.04)	229	4.83	0.028
C6	0.33 (0.05)	351	0.41 (0.06)	229	1.23	0.268
C7	1.35 (0.04)	351	1.40 (0.05)	229	0.69	0.407
IN < 5 (%)	6.40 *	27	10.20 *	31	-	0.039
IN > 5 (%)	93.60 *	398	89.90 *	272		
UP < 5 (%)	81.90 *	348	79.90 *	242	-	0.278
UP > 5 (%)	18.10 *	77	20.10 *	61		

Data are presented as mean ± standard error, adjusted for age and sex. Statistical analysis: ANCOVA, covariate = age and sex; * Data presented as percentage frequency; Statistical analysis: Chi-square test. Confidence interval:95%; level of significance: *p* < 0.05. Abbreviations*:* SE: standard error; *n*: absolute number; GNCOV: group not infected with COVID-19; GCOV: group infected with COVID-19; BMI: body mass index; UE: uncontrolled eating; CR: cognitive restriction; EE: emotional eating; BAI: score referring to Beck’s anxiety scale; PSQI: score referring to Pittsburgh sleep quality index; C1: subjective sleep quality component; C2: latency component; C3 duration component; C4: efficiency component; C5: sleep disorders component; C6: sleep medication use component; C7: daytime dysfunction component; IN: *in natura* or minimally processed; UP: ultra-processed.

**Table 2:** Relationship of eating pattern with eating behavior, anxiety level, and sleep quality in individuals diagnosed with COVID-19.

Ultraprocessed							*In natura*					
	<5	*n*	>5	*n*	F	*p*	<5	*n*	>5	*n*	F	*p*
UE	33.80 (1.48)	186	37.71(3.12)	42	1.28	0.259	36.74 (4.44)	21	34.29 (1.40)	207	0.28	0.601
EE	40.10 (2.02)	188	42.93 (4.18)	44	0.37	0.545	44.77 (6.09)	21	40.23 (1.90)	211	0.51	0.48
CR	46.42 (1.69)	188	40.56 (3.54)	43	2.22	0.138	49.21 (5.12)	21	44.94 (1.60)	210	0.63	0.427
BAI	11.67 (0.74)	186	14.71 (1.56)	42	3.11	0.079	15.01 (2.22)	21	11.95 (0.70)	207	1.73	0.190
PSQI	6.68 (0.27)	161	8.10 (0.62)	31	4.28	0.040	5.50 (0.89)	18	7.05 (0.26)	174	3.173	0.077

Data are presented as mean ± standard error, adjusted for age and sex. Statistical analysis: ANCOVA, covariate = diabetes mellitus, arterial hypertension, time of onset of symptoms of COVID-19, age and sex. Confidence interval: 95%; level of significance: *p* < 0.05. Abbreviations: *n*, absolute number; UE, uncontrolled eating; CR, cognitive restriction; EE, emotional eating; BAI, Beck Anxiety Scale; PSQI, Pittsburgh Sleep Quality Index.

**Table 3:** Relationship of anxiety level with eating pattern of individuals infected with COVID-19.

Level of Anxiety Symptoms									
	Minimum (*n* = 162)		Light (*n* = 83)		Moderate (*n* = 32)		Severe (*n* = 26)		
	%	*n*	%	*n*	%	*n*	%	*n*	*p*
UP < 5	81.50	132	84.30	70	68.80	22	69.20	18	0.134
UP > 5	18.50	30	15.70	13	31.30	10	30.80	8	
IN < 5	10.50	17	7.20	6	12.50	4	15.40	4	0.624
IN > 5	89.50	145	92.80	77	87.50	28	84.60	22	

Data presented as percentage frequency and absolute number; Statistical analysis: chi-square. Confidence interval: 95%, significance level: *p* < 0.05. Abbreviations: *n*, absolute number; IN, *in natura*; UP, ultra-processed.

**Table 4:** Relationship of BMI with eating behavior, anxiety level and sleep quality of individuals infected with COVID-19.

					BMI					
	Low Weight		Eutrophy		Overweight		Obesity			
	Mean (SE)	*n*	Mean (SE)	*n*	Mean (SE)	*n*	Mean (SE)	*n*	f	*p*
UE	30.56 (5.88) ^ab^	11	29.51 (1.68) ^a^	137	40.75 (2.97) ^bc^	43	47.02 (3.42) ^c^	37	8.39	<0.001
EE	26.52 (7.88) ^a^	11	33.34 (2.22) ^a^	141	52.37 (3.99) ^ab^	43	58.99 (4.59) ^b^	37	11.88	<0.001
CR	14.12 (6.79) ^a^	11	45.15 (1.92) ^b^	140	49.74 (3.44) ^b^	43	50.13 (3.95) ^b^	37	0.79	<0.001
BAI	8.85 (2.98) ^ab^	11	10.05 (0.85) ^a^	137	15.69 (1.51) ^bc^	43	17.27 (1.73) ^c^	37	6.67	<0.001
PSQI	7.74 (1.10) ^ab^	10	6.25 (0.32) ^b^	117	8.47 (0.58) ^a^	36	7.32 (0.69) ^a^	29	4.08	0.008

Data are presented as mean ± standard error, adjusted for age and sex. Statistical analysis: ANCOVA, covariate = diabetes mellitus, arterial hypertension, time of onset of symptoms of COVID-19, age and sex; Sidak post hoc test. Different letters: significant difference (*p* < 0.05). Abbreviations*:* SE, standard error; *n*, absolute number; BMI, body mass index; UE, uncontrolled eating; CR, cognitive restriction; EE, emotional eating; BAI, Beck’s anxiety scale; PSQI, Pittsburgh Sleep Quality Index.

**Table 5:** Relationship of the duration of symptoms with eating behavior and anxiety level of individuals infected with COVID-19.

		Unstandardized Coefficients		Standardized Coefficients			
	Model	B	SE	β	t	*p*	r^2^
UE	>15 days	8.441	3.276	0.164	2.576	0.011	0.027
EE	>15 days	6.955	4.635	0.096	1.501	0.135	0.009
CR	>15 days	5.412	3.622	0.096	1.494	0.136	0.009
BAI	>15 days	4.390	1.645	0.169	2.668	0.008	0.029

Statistical analysis: multiple regression analysis. Abbreviations: SE, standard error; UE, uncontrolled eating; CR, cognitive restriction; EE, emotional eating; BAI, Beck’s anxiety scale.

**Table 6:** Relationship of onset of symptoms with eating behavior and anxiety level of individuals infected with COVID-19.

		Unstandardized Coefficients		Standardized Coefficients			
	Model	B	SE	β	t	*p*	r^2^
UE	2–3 months	2.668	5.469	0.029	0.488	0.626	0.008
	4–7 months	−1.008	2.002	−0.031	−0.504	0.615	
	8–11 months	−0.037	1.114	−0.002	−0.033	0.974	
	>12 months	−0.950	0.744	−0.083	−1.277	0.203	
EE	2–3 months	4.063	7.726	0.032	0.526	0.599	0.016
	4–7 months	1.756	2.828	0.038	0.621	0.535	
	8–11 months	1.007	1.574	0.041	0.640	0.523	
	>12 months	−1.491	1.051	−0.091	−1.418	0.157	
CR	2–3 months	−1.788	6.055	−0.018	−0.295	0.768	0.006
	4–7 months	−2.161	2.216	−0.061	−0.975	0.330	
	8–11 months	0.727	1.234	0.038	0.589	0.556	
	>12 months	0.123	0.824	0.010	0.149	0.881	
BAI	2–3 months	−1.055	2.858	−0.022	−0.369	0.712	0.015
	4–7 months	−0.494	1.046	−0.029	−0.473	0.637	
	8–11 months	−0.774	0.582	−0.085	−1.330	0.185	
	>12 months	−0.773	0.389	−0.128	−1.986	0.048	

Statistical analysis: multiple regression analysis. Abbreviations: SE, standard error; UE, uncontrolled eating; CR, cognitive restriction; EE, emotional eating; BAI, Beck’s anxiety scale.

## Data Availability

The datasets generated and/or analyzed during the current study are not publicly available but are available from the corresponding author upon reasonable request.

## Supplementary Materials

The following supporting information can be downloaded at: https://www.mdpi.com/article/10.3390/nu15092148/s1, Figure S1. Flowchart of participants selected for the study.; Table S1. Sociodemographic, anthropometric, dietary intake and lifestyle characteristics during the COVID-19 pandemic; Table S2. Effect of taste and smell dysfunctions on anthropometric data, eating behavior, anxiety level, and sleep quality in adult individuals residing in Brazil.
