# Supplementary material for: Impact of Final Phase Social Isolation and the COVID-19 Pandemic on Eating Behavior, Sleep Quality, and Anxiety Level
Source: Nutrients. 2023 Apr 29;15(9):2148. doi: 10.3390/nu15092148 (PMC10181063; doi:10.3390/nu15092148)
Supplement: Supplementary file 1 [file nutrients-15-02148-s001.zip › nutrients-2279720-supplementary.pdf]

SUPPLEMENTARY MATERIAL

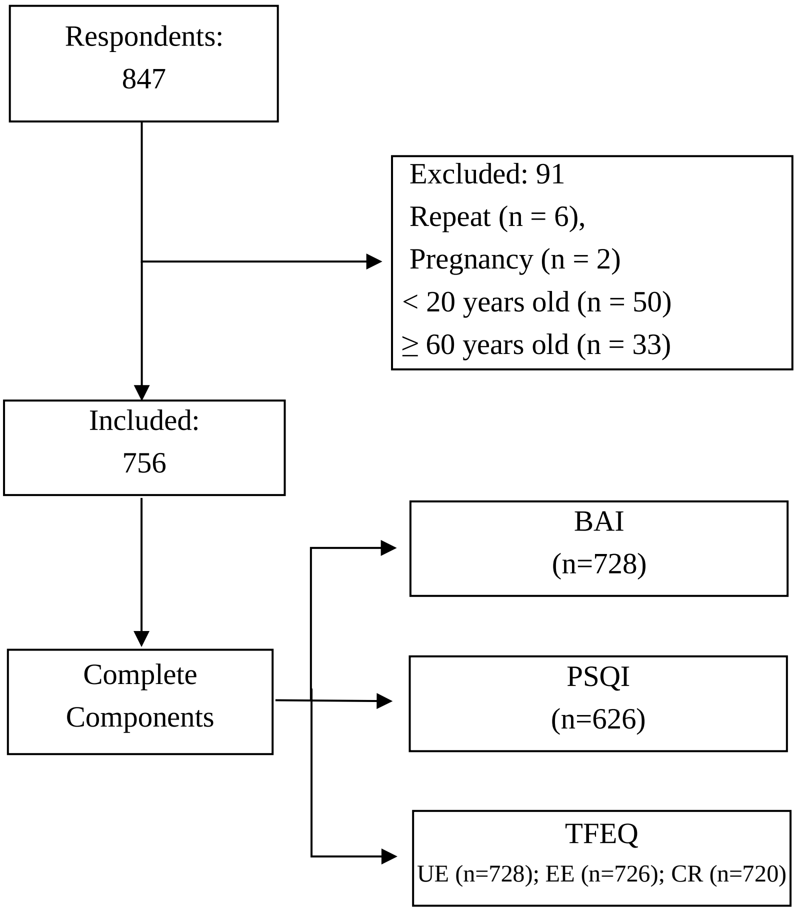

Figure S1. Flowchart of participants selected for the study.

BAI, Beck Anxiety Scale; PSQI, *Pittsburgh Sleep Quality Index;* TFEQ-21, *Three-Factor Eating Questionnaire*; UE, uncontrolled eating; EE, emotional eating; CR, cognitive restriction.

Table S1. Characterization of the participants **(n=756)**

		Total		COVID					
				**No**		**Yes**		**Don't know**	
		n	%	n	%	n	%	n	%
Region (617)	Midwest	305	49,4%	166	26,9%	129	20,9%	10	1,6%
	North	9	1,5%	2	0,3%	5	0,8%	2	0,3%
	Northeast	45	7,3%	28	4,5%	15	2,4%	2	0,3%
	Southeast	215	34,8%	118	19,1%	86	13,9%	11	1,8%
	South	43	7,0%	19	3,1%	24	3,9%	0	0,0%
Sex (756)	Male	138	18,3%	86	11,4%	47	6,2%	5	0,7%
	Female	618	81,7%	339	44,8%	256	33,9%	23	3,0%
Level of Education (755)	Fundamental	6	0,8%	4	0,5%	2	0,3%	0	0,0%
	Medium	279	37,0%	146	19,3%	121	16,0%	12	1,6%
	Superior	450	59,6%	262	34,7%	174	23,0%	14	1,9%
	Other	20	2,6%	12	1,6%	6	0,8%	2	0,3%
Currently Working (756)	No	266	35,2%	143	18,9%	111	14,7%	12	1,6%
	Yes	478	63,2%	278	36,8%	185	24,5%	15	2,0%
	Do not wantto answer	12	1,6%	4	0,5%	7	0,9%	1	0,1%
Family income (751)	Less than 1 MW	11	1,5%	6	0,8%	3	0,4%	2	0,3%
	1MW	17	2,3%	7	0,9%	8	1,1%	2	0,3%
	From 2 to 4 MW	218	29,0%	107	14,2%	97	12,9%	14	1,9%
	From 5 to 7 MW	166	22,1%	107	14,2%	54	7,2%	5	0,7%
	More than 8 MW	287	38,2%	170	22,6%	114	15,2%	3	0,4%
	Do not wantto answer/other	52	6,9%	27	3,6%	23	3,1%	2	0,3%

Data are expressed as absolute numbers (n) and percentage frequencies (%). *Abbreviations:* MW: minimum wage.

Table S2. Effect of taste and smell dysfunctions on anthropometric data, eating behavior, anxiety level, and sleep quality in adult individuals residing in Brazil.

Variables	GCOV with taste dysfunctions		GCOV Asymptomatic			
	**Mean (SE)**	**n**	**Mean (SE)**	**n**	**f**	**p**
Pre-pandemic weight	66.97 (1.37)	130	67.16 (1.55)	101	0.01	0.926
BMI pre-pandemic	24.57 (0.45)	130	24.62 (0.52)	101	0.01	0.946
Current weight	67.87 (1.33)	130	67.49 (1.52)	101	0.03	0.854
Current BMI	24.88 (0.43)	130	24.74 (0.49)	101	0.04	0.841
Weight variation	0.91 (0.76)	130	0.33 (0.87)	101	0.24	0.624
UE	36.64 (1.79)	130	31.61 (2.05)	101	3.27	0.072
EE	42.53 (2.46)	130	36.99 (2.81)	101	2.93	0.088
CR	42.82 (2.07)	130	48.71 (2.37)	100	3.37	0.068
BAI	11.94 (0.93)	130	12.78 (1.06)	101	0.35	0.558
PSQI	6.73 (0.35)	106	7.15 (0.39)	85	0.64	0.425
C1	1.13 (0.08)	106	1.25 (0.09)	85	0.95	0.332
C2	1.40 (0.09)	106	1.50 (0.11)	85	0.39	0.535
C3	0.71 (0.07)	106	0.91 (0.08)	85	3.64	0.058
C4	0.47 (0.09)	106	0.54 (0.09)	85	0.28	0.599
C5	1.23 (0.05)	106	1.16 (0.06)	85	0.90	0.343
C6	0.35 (0.08)	106	0.37 (0.09)	85	0.02	0.886
C7	1.43 (0.08)	106	1.44 (0.09)	85	0.03	0.955
IN < 5	6.90*	10	13.90*	16	-	0.050
IN > 5	93.10*	134	86.10*	99	-	0.050
UP < 5	79.90*	115	81.70*	94	-	0.414
UP > 5	20.10*	29	18.30*	21	-	0.414

Variables	GCOV with smell disfunction		GCOV Asymptomatic			
	Mean (SE)	N	Mean (SE)	n	f	P
Pre-pandemic weight	67.23 (1.35)	134	66.81 (1.61)	97	0.04	0.845
BMI pre-pandemic	24.65 (0.45)	134	24.51 (0.53)	97	0.035	0.851
Current weight	67.52 (1.32)	134	67.95 (1.56)	97	0.04	0.839
Current BMI	24.72 (0.43)	134	24.96 (0.50)	97	0.13	0.724
Weight variation	0.30 (0.75)	134	1.15 (0.89)	97	0.50	0.479
UE	34.98 (1.79)	134	33.69 (2.12)	97	0.21	0.651
EE	42.25 (2.45)	134	38.48 (2.91)	97	0.93	0.337
CR	43.89 (2.06)	134	47.45 (2.46)	96	1.16	0.283
BAI	11.52 (0.92)	134	13.39 (1.09)	97	1.63	0.203
PSQI	6.93 (0.35)	110	6.90 (0.41)	85	0.001	0.970
C1	1.11 (0.08)	110	1.28 (0.09)	85	0.18	0.186
C2	1.47 (0.09)	110	1.41 (0.11)	85	0.15	0.702
C3	0.73 (0.07)	110	0.89 (0.08)	85	2.05	0.153
C4	0.49 (0.09)	110	0.51 (0.10)	85	0.02	0.884
C5	1.24 (0.05)	110	1.14 (0.06)	85	1.238	0.267
C6	0.44 (0.08)	110	0.25 (0.09)	85	2.065	0.152
C7	1.45 (0.08)	110	1.42 (0.09)	85	0.03	0.861
IN < 5	3.10*	8	6.90*	18	-	0.005
IN > 5	53.30*	138	36.7*	96	-	0.005
UP < 5	82.20*	120	78.70*	89	-	0.295
UP > 5	17.80*	26	21.20*	24	-	0.295

Data are presented as mean ± standard error, adjusted for age and sex. Statistical analysis: ANCOVA, covariate = diabetes mellitus, arterial hypertension, time of onset of symptoms of Covid-19, age and sex; *Data presented as percentage frequency; Statistical analysis: chi-square test. Confidence interval: 95%, level of significance: $P < 0.05$. *Abbreviations:* SE, standard error; n, absolute number; GNCV: group not infected with COVID-19; GCOV: group infected with Covid-19; BMI: body mass index; UE: uncontrolled eating; CR: cognitive restriction; EE: emotional eating; BAI: score referring to Beck's anxiety scale; PSQI: score referring to *Pittsburgh Sleep Quality Index*; C1: subjective sleep quality component; C2: latency component; C3: duration component; C4: efficiency component; C5: sleep disorders component; C6: sleep medication use component; C7: daytime dysfunction component; IN: *in natura,* UP: ultra-processed.
